# Isolated Unilateral Posterior Sacroiliac Dislocation With an Intact Anterior Pelvic Ring: Case Report of an Unusual Injury Pattern

**DOI:** 10.1155/cro/9605757

**Published:** 2026-06-22

**Authors:** Leonardo Comerlatto, Tiago Zimerman, Natália Henz Concatto, Vincenzo Giordano, Leandro de Freitas Spinelli, Marcelo Faria Silva

**Affiliations:** ^1^ Department of Orthopedics and Traumatology, Porto Alegre Emergency Hospital, Porto Alegre, Rio Grande do Sul, Brazil; ^2^ Department of Orthopedics and Traumatology, Federal University of Health Sciences of Porto Alegre, Porto Alegre, Rio Grande do Sul, Brazil, ufcspa.edu.br; ^3^ Department of Orthopedics and Traumatology, Moinhos de Vento Hospital, Porto Alegre, Rio Grande do Sul, Brazil, hospitalmoinhos.org.br; ^4^ Department of Orthopedics and Traumatology, Mae de Deus Hospital, Porto Alegre, Rio Grande do Sul, Brazil; ^5^ Brazil Institute of Health Technologies, Rio de Janeiro, Rio de Janeiro, Brazil; ^6^ Department of Orthopedics and Traumatology, Miguel Couto Municipal Hospital, Rio de Janeiro, Rio de Janeiro, Brazil

**Keywords:** case report, pelvic fracture, posterior sacroiliac dislocation, trauma

## Abstract

Unstable pelvic ring injuries usually present with pubic symphysis and/or pubic rami disruption, in association with fractures or fracture‐dislocations affecting the posterior iliac, sacroiliac joint, and/or the sacrum. Involvement of the strong posterior osteoligamentous complex with an intact anterior pelvic ring is extremely rare, with only a limited number of cases previously documented. We report the case of a 15‐year‐old male who sustained high‐energy trauma after being struck in the back by a heavy metal structure. He presented with a unilateral posterior sacroiliac dislocation and an intact anterior pelvic ring, which is believed to represent a previously unreported injury. The patient underwent closed reduction with percutaneous fixation using a 7.0 mm partially threaded cannulated iliosacral screw. This case highlights an unusual pelvic injury pattern not fully addressed by current classification systems and emphasizes the importance of carefully assessing anterior ring integrity in high‐energy posterior pelvic trauma.

## 1. Introduction

Mechanically unstable pelvic ring injuries (PRIs) usually present with pubic symphysis and/or pubic rami disruption, in association with fractures or fracture‐dislocations affecting the posterior pelvic ring (posterior iliac, sacroiliac [SI] joint, and/or sacrum) [1]. Despite the mechanism, PRIs typically occur along a spectrum in which the weaker anterior structures are initially affected. Pelvic global stability depends upon the integrity of the posterior osteoligamentous complex [2], and based on the energy involved, the stronger posterior anatomic structures are gradually compromised.

Posterior PRIs that occur without anterior involvement are rare, of uncertain mechanism, and barely comprehended by existing classification systems [3–5]. After a comprehensive literature review, eight case reports of bilateral SI joint posterior dislocations or fracture‐dislocations [6–13] were identified, which are sometimes described as *intrapelvic protrusion of the sacrum*. To our knowledge, only one case of unilateral complete posterior pelvic ring injury without anterior involvement has been reported, in which the posterior injury was an anterior SI joint dislocation [14].

We present the case of an adolescent patient who sustained a unilateral posterior SI joint dislocation with an intact anterior pelvic ring, which is believed to represent a previously unreported injury.

## 2. Case Presentation

**Figure 1:**
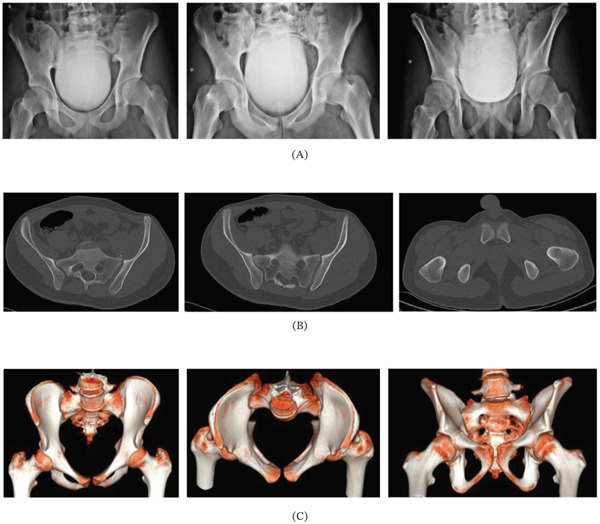
Preoperative imaging. (A) Preoperative pelvic radiographs in the anteroposterior (AP), inlet, and outlet views. (B) Axial CT scan images demonstrating the first and second sacral segments and the pubic symphysis. (C) Three‐dimensional CT reconstruction of the pelvis.

A 15‐year‐old male was admitted to a Level 1 Trauma Center after being struck in the back by a heavy metal structure that fell from a crane. He had no relevant medical, family, or psychosocial history and no history of pelvic trauma, surgery, or other relevant interventions. The patient presented to the emergency department hemodynamically stable, awake and referring thoracic, abdominal, and low back pain. Initial assessment and imaging demonstrated left side multiple rib fractures, myocardial and pulmonary contusion, a minimal pneumothorax, Grade III splenic laceration, and a PRI. Pelvis radiographs and computed tomography (CT) scans revealed a posterior dislocation of the left SI joint without associated anterior pelvic ring injury (Figure 1). It was also identified a left side L1 transverse process fracture and a minor left side incomplete sacral ala fracture. Thoracic and abdominal trauma was considered for nonoperative treatment, and the patient was admitted to the pediatric intensive care unit. Preoperative ambulatory status could not be assessed because the patient remained on strict bed rest due to his injuries. Due to its atypical presentation, the PRI was scrutinized. The patient confirmed that the impact occurred in a posterior‐to‐anterior direction and solely reported pain in the left posterior SI joint area, which could be exacerbated by direct pressure applied by the examiner, and denied pain in the anterior pelvic ring, including the pubic symphysis, even upon deep palpation. Lumbar and posterior pelvic region inspection did not reveal any clinically significant soft‐tissue or cutaneous findings, despite the high‐energy mechanism involved. Neurological examination of the lower extremities was normal, with preserved motor and sensory function. No tenderness, instability, or pain was elicited over the anterior pelvic structures, even under direct palpation. A complete and displaced posterior ring injury in association with pelvic global deformity (left hemipelvis apparently rotated over an intact pubic symphysis) was the factor guiding the indication for surgical treatment, conducted on hospital Day 5.

**Figure 2:**
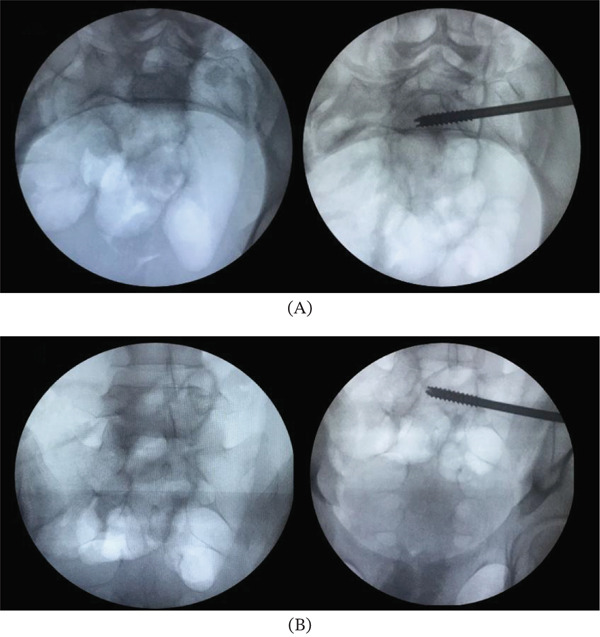
Intraoperative fluoroscopic images. Fluoroscopic (A) inlet and (B) outlet views obtained before and after iliosacral fixation.

**Figure 3:**
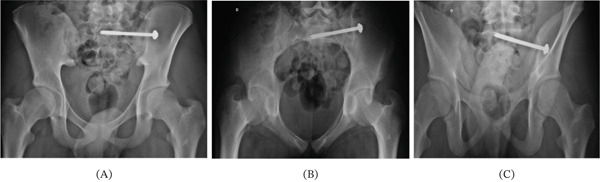
Postoperative radiographs. Postoperative pelvic radiographs in the (A) anteroposterior (AP), (B) inlet, and (C) outlet views.

**Figure 4:**
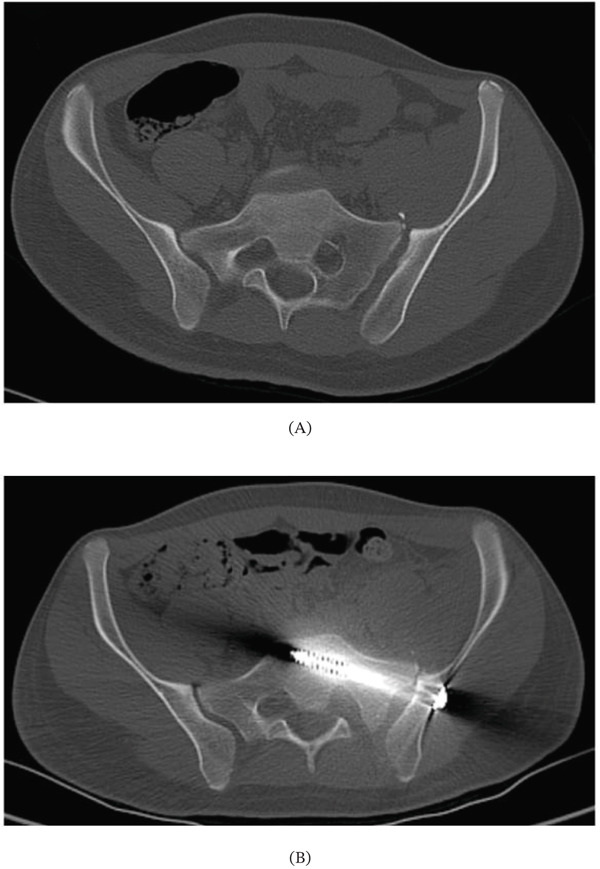
Postoperative CT scan. Comparative axial CT images of the upper sacral segment obtained (A) preoperatively and (B) after iliosacral fixation.

The patient was positioned supine and underwent examination under anesthesia (EUA) according to previously described method [15]. Internal rotation stress testing was performed with the lower extremities in adduction and internal rotation while simultaneous compression was applied through both greater trochanters. External rotation testing was then performed using the frog‐leg position with an abduction force applied to both knees. Vertical instability was assessed with push–pull maneuvers, consisting of longitudinal traction applied to one lower extremity while simultaneous axial loading was applied to the contralateral limb; the maneuvers were subsequently repeated with the opposite extremities. No displacement was identified in the anterior pelvic ring during any of the stress tests. However, the left posterior pelvic ring demonstrated instability and displacement during all maneuvers performed. Femoral skeletal traction was applied, resulting in satisfactory closed reduction. Residual SI joint gap was compressed with a 70‐mm length, 7.0‐mm diameter, partially threaded cannulated iliosacral screw with a 32‐mm thread length, percutaneously inserted under fluoroscopy across the left SI joint into the upper sacral segment (S1). (Figure 2). Postoperative images (Figures 3 and 4) showed correction of the left hemipelvic deformity and substantial restoration of left SI joint congruity. Even though a small residual posterior incongruity was present, the reduction was considered acceptable because there was no gross residual displacement on postoperative images, the anterior pelvic ring remained congruent, and stable iliosacral fixation was achieved. The patient had an uneventful immediate postoperative period and was discharged on Day 10 of hospitalization in stable clinical condition. At discharge, pain was controlled, and no early surgical‐related complications, such as wound or fixation‐related complications, were identified. He was instructed to follow an initial 6‐week period of protected weight‐bearing on the left side, while early sitting and mobilization were encouraged as tolerated, followed by assisted gait training and continued crutch use until gait was safe, comfortable, and painless. The patient presented at the 8‐week follow‐up, ambulating without assistance and asymptomatic, with no clinical evidence of early loss of stability or implant‐related symptoms. The patient did not return for further appointments despite being informed, along with his family, about the need for long‐term follow‐up and future screw removal.

## 3. Discussion

**Figure 5:**
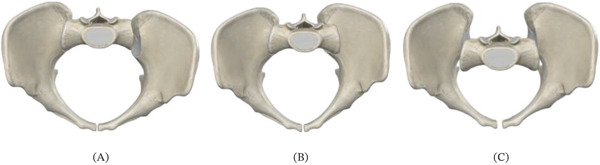
Previously described possible types of posterior pelvic ring dislocations (or fracture‐dislocations) with an intact anterior pelvic ring. (A) Type I, unilateral anterior sacroiliac joint dislocation. (B) Type II, unilateral posterior sacroiliac joint dislocation. (C) Type III, bilateral posterior sacroiliac joint dislocation.

We reported a case of an isolated unilateral posterior SI dislocation with an intact anterior pelvic ring. To our knowledge, no previous reports have described this injury pattern, indicating this is a rare and unique injury. Because we had the precise information that our patient was compressed by a heavy structure that directly impacted his back, it was possible to identify similarities with other posterior isolated injuries, which indeed resemble a posteroanterior compression mechanism. Previous reports are summarized in Table 1. Among previous reports, LaFollette et al. [7], Wright et al. [10], Hungerer et al. [12] and Brown et al. [13] presented patients of similar age range, all 16 or 17 years old, which may suggest that certain skeletal characteristics in this population could favor its occurrence. It is important to recognize that pelvic injuries in adolescents may not conform to either adult or pediatric characteristics because of the transitional characteristics of this age group. Except for one case that resulted from an equestrian accident, all other injuries reported in the literature resulted from markedly high‐energy trauma, including falls from heights over 20 feet. Marcus and Hansen [6], LaFollette et al. [7], Nagi et al. [8], Stevens et al. [9], Loupasis et al. [11], and Hungerer et al. [12] were able to specify that the patients sustained a severe impact to the back or landed directly on the buttocks, which may be indicative of the force vectors required to cause this unusual injury pattern. It was suggested by Nagi et al. [8], Wright et al. [10], Loupasis et al. [11], and Brown et al. [13] that this injury may have been caused by a direct posterior‐to‐anterior compressive force on the sacrum. Interestingly, Tile [16] included the bilateral SI dislocation with an intact anterior arch as a C3 variant, which was usually seen in young females, caused by a crush with the patient hyperflexed. Bouguennec et al. [14] reported an isolated anterior unilateral SI dislocation without involvement of anterior structures. The patient was a 20‐year‐old male who was a backseat passenger ejected from a motor vehicle in a high‐energy accident. Although the case we reported is also unilateral, it differs from that reported by Bouguennec et al. Their patient had an anterior unilateral SI dislocation after a motor vehicle accident, whereas our patient sustained a posterior unilateral SI dislocation after a direct posterior‐to‐anterior impact. Additionally, the present case involved a younger patient, 15 years old, compared with 20 years old. Since there are no similar reports, unilateral injuries involving the posterior pelvic ring with intact anterior structures seem to be an even more uncommon situation. Nevertheless, the treatment principle remains similar to other posterior SI dislocations, as restoring posterior stability through anatomical reduction and fixation is essential to prevent long‐term impairments. Based on previously reported cases and adding the pattern described here, Figure 5 demonstrates possible types of posterior pelvic ring dislocations or fracture‐dislocations without concomitant anterior pelvic ring injury.

**Table 1:** Reported sacroiliac dislocations without anterior pelvic ring disruption and comparison with the present case.

Study	Age/sex	Mechanism	Injury pattern/direction	Anterior ring status	Fixation/treatment	Outcome
Marcus and Hansen, 1984	34/M	Struck across the posterior sacrum by a hydraulic lift	Complete bilateral sacroiliac fracture‐dislocation with displacement of the sacrum into the pelvis; sacrum displaced anteriorly and inferiorly; fracture entered both SI joints and involved the iliac articular surfaces, with comminution on the right	No associated pelvic ring disruption described	ORIF through posterior bilateral SI approaches; overhead traction applied directly to the sacrum; cancellous screws through plates between each PSIS and sacrum; Steinmann pins and figure‐of‐eight tension‐band wiring	Anatomical alignment/healing at 6 weeks; weight‐bearing started at 6 weeks. At 7.5 months, slight right‐sided limp, no pain, painful ejaculation resolved; then lost to follow‐up
LaFollette et al., 1986	17/F	Thrown from a horse, landing on both ischial tuberosities	Bilateral sacroiliac fracture‐dislocation/dislocation with intrapelvic displacement of the sacrum	No anterior pelvic ring injury described	Nonoperative management with active and active‐assisted mobilization; no reduction attempt and no skeletal traction	Walked with walker by 10 days; discharged using crutches in 3rd week; crutches discontinued by 12 weeks. At 7 months, fully recovered and back to horseback riding/sports. At 5.5 years, asymptomatic with normal exam; SI fusion in deformity and 3–4 cm height loss
Nagi et al., 1993	20/F	Fell from the pillion seat of a motorcycle and landed squarely on her buttocks	Bilateral fracture‐dislocation of the sacroiliac joints with intrapelvic protrusion of the sacrum	Rest of the pelvic ring intact	Longitudinal skeletal traction through both tibial tubercles. Approximately 80% reduction at 2 weeks; traction weight progressively increased; mobilization started at 8 weeks when traction was discontinued	At 12 weeks, walked painlessly with crutches and had no clinical instability. At 1 year, fully ambulatory with adequate hip and spine motion; 2 cm height loss; SI joints stable in a partially displaced position
Stevens et al., 1997	27/M	Fell 20 feet/6.1 meters from a window, landing on his back and right arm	Bilateral fracture‐dislocation of the SI joints with displacement of the sacrum into the pelvis; CT showed widened SI joints, inferior dislocation of the sacrum, anterior displacement of the sacrum, and fracture of the posterior left ilium	No anterior pelvic fracture or disruption	Posterior open reduction and internal fixation	Restoration of the pelvic ring produced immediate pain reduction; detailed long‐term outcome not reported
Wright et al., 2004	17/M	High‐speed motor vehicle collision; unrestrained driver struck a tree at 85 mph	Pure bilateral SI joint dislocation with slight pelvic intrusion of the sacrum	No anterior pelvic ring injury	Bilateral anterior ORIF; each SI joint reduced and stabilized with two contoured 4.5‐mm reconstruction plates	Full weight‐bearing without pain at 3 months. At 4 months, no pain, weakness, or limp
Loupasis et al., 2005	27/M	Fell from the second floor (6.5 meters), landing on buttocks	Pure bilateral SI dislocation with intrapelvic protrusion of the sacrum; 3D CT showed anterior and inferior/downward displacement of the sacrum relative to the iliac bones	No acetabular fracture, pubic rami fracture, or symphyseal disruption	Initial bilateral distal femoral traction was ineffective; delayed ORIF at 21 days through anterior approach; fixation with two 3‐hole, 3.5‐mm DCP plates; 1 cm residual displacement	At 6 months, walked with a limp. At 24 months, returned to work; mild left L5 causalgia and left buttock discomfort with excessive activity; mild left ankle weakness; SI fusion and extensive heterotopic ossification of posterior SI ligaments
Hungerer et al., 2008	16/M	Uncontrolled ski‐jump landing directly on buttocks	Isolated bilateral SI dislocation; vertical shear pattern; iliac portion of SI joint displaced >2 cm cranially; complete ligament disruption and avulsion at sacral apophysis	No anterior ring fracture or symphyseal disruption	Initial dorsal iliolumbar transfixation; postoperative CT showed persistent malreduction; revised with dorso‐ventro‐dorsal approach: dorsal release, anterior bilateral double plating, and retightening of iliolumbar fixation	Uneventful recovery; discharged after 4 weeks to rehabilitation; gradual loading; unrestricted daily activities by 8 weeks; no pain or limitations at 8 weeks; sports restricted for 6 months
Bouguennec et al., 2012	20/M	High‐energy motor vehicle accident; backseat passenger ejected	Isolated unilateral right anterosuperior SI dislocation	No anterior pelvic ring injury	External reduction failed; open reduction through small paravertebral approach; no SI fixation because of hemodynamic instability	No secondary displacement at 1 and 2 months. At 24 months, patient remained in neurovegetative state; CT showed congruent SI joint, persistent gap <1 cm, and calcifications
Brown et al., 2023	16/M	Dirt bike versus motor vehicle collision at 55 mph; head‐on collision	Isolated bilateral ligamentous SI dislocation; MRI showed posterior and superior displacement of both ilia relative to the sacrum with rupture of the SI ligaments	No anterior pelvic ring injury	ORIF through lateral window of ilioinguinal approach; bilateral traction, bilateral SI screws, right S1 fully threaded screw and left S2 partially threaded screw	At 3 months, able to jump without pain and satisfied with outcome; radiographs showed stable fixation with slight residual left SI malreduction
Current case	15/M	Direct posterior‐to‐anterior impact	Isolated unilateral posterior SI dislocation	Intact anterior pelvic ring	Closed reduction + percutaneous 7.0‐mm partially threaded cannulated iliosacral screw	Stable discharge on postoperative Day 10; pain controlled; no early complications. At 8 weeks, the patient was asymptomatic and ambulating without assistance, with no clinical evidence of early loss of stability or implant‐related symptoms; subsequently lost to follow‐up.

*Note:* Direction was reported according to the terminology used by each article. In bilateral injuries, some authors described displacement of the sacrum relative to the iliac bones, whereas others described displacement of the ilia relative to the sacrum.

Abbreviations: ORIF, open reduction and internal fixation; SI, sacroiliac.

In this unusual scenario, confirming anterior pelvic ring integrity was a key point for inferring possible force vectors involved, which ultimately guides reduction and fixation strategies. In addition to the available imaging data, this definition was based on the absence of pain or other clinical features over anterior structures, along with negative EUA findings. Although not available at the institution where this study was conducted, magnetic resonance imaging could have been useful once it has been shown to aid in the detection of occult pelvic fractures and pubic symphysis injuries [17, 18].

This injury pattern is difficult to classify with current pelvic ring classification systems [3–5]. The Young–Burgess classification is based on the presumed force vector and generally associates posterior pelvic ring instability with anterior ring disruption. However, in our case, posterior SI instability occurred with an intact anterior pelvic ring, making it difficult to fit within the categories. Similarly, the Tile classification, which is based on pelvic stability, does not specifically account for unilateral posterior SI dislocation with an intact anterior ring. Therefore, these systems do not adequately describe the current injury morphology or the likely mechanism of injury.

**Figure 6:**
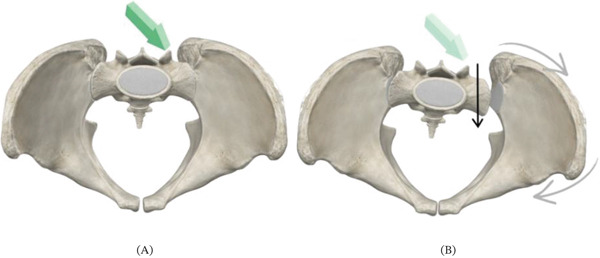
Proposed mechanism resulting in an isolated unilateral posterior sacroiliac joint dislocation. High energy, (A) direct posterior‐to‐anterior impact to the sacrum and left side posterior iliac bone (green arrow), (B) forcing the sacrum anteriorly (black arrow) and creating a rotational deformity in the left hemipelvis (gray arrows), over an intact anterior pelvic ring. Subtle variations in the force vector may cause the other previously described injury patterns.

Considering that preexisting classification systems do not encompass this specific pattern, and in line with previous reports, a reasoned analysis of the clinical history and imaging findings allows the proposal of an injury mechanism. We hypothesize that this injury resulted from a high‐energy posterior‐to‐anterior force vector being applied directly to the sacrum, resulting in asymmetric forced anterior translation of the sacrum relative to the posterior iliac. This led to a complete loss of congruence of the left SI joint, with the ilium displaced posteriorly in relation to the sacrum. Simultaneously, the relatively flexible pubic symphysis remained intact, serving as an anterior fulcrum over which some degree of rotation of the left hemipelvis could have occurred. Even though the CT did not show gross anterior or posterior displacement, the presence of complete SI joint incongruence with rotational deformity during stress testing strongly suggests disruption of the posterior SI ligamentous complex. Despite the injury mechanism, unreduced and/or unstable SI joints are typically associated with poor long‐term clinical outcomes [4, 19–21], making anatomic reduction and stable fixation appropriate for this patient. Recent studies have shown that percutaneous iliosacral screw fixation is a safe and effective method for managing unstable posterior PRIs [22] and that closed reduction combined with percutaneous screw fixation provides satisfactory functional results, supporting minimally invasive stabilization as a reliable and effective treatment when anatomical reduction is achieved. [21]. A schematic representation of the proposed injury pattern is shown in Figure 6.

Although instability was identified during stress maneuvers, only static intraoperative fluoroscopic images demonstrating a completely displaced injury of the left posterior pelvic ring were documented. Dynamic fluoroscopic images obtained during the stress examination were not recorded, which represents a limitation of the present case report. Although anterior ring stability was assessed intraoperatively with real‐time fluoroscopy, representative dynamic images could not be retrieved because the institution′s fluoroscopy equipment did not support image archiving. Residual displacement was not formally quantified in millimeters at the time of treatment, which represents a limitation of this retrospective case report. Although the patient was asymptomatic and ambulating without assistance at 8 weeks, he was subsequently lost to follow‐up despite being oriented regarding the importance of continued surveillance. Therefore, long‐term clinical and radiographic assessments could not be performed, which limits the ability to make definitive conclusions regarding maintenance of pelvic stability, late pain, gait outcomes, implant integrity, potential late complications, and long‐term functional results. Consequently, conclusions regarding long‐term outcomes should be interpreted with caution and are limited to the early postoperative period. Despite this drawback, this case deserves consideration given the rarity of this injury pattern, and the data provided may contribute to broadening current understanding regarding atypical posterior pelvic ring disruption.

In conclusion, this case emphasizes that current concepts and classification systems do not account for posterior PRIs with intact anterior structures. Likely due to the rarity of this injury, no anatomical or biomechanical model has been developed to replicate this mechanism. It is important for orthopedic surgeons specializing in pelvic trauma to continue publishing similar cases and pursuing basic research to improve understanding of this atypical injury pattern.

## Funding

No funding was received for this manuscript.

## Disclosure

The study was presented as an e‐poster at the XXVII Brazilian Orthopedic Trauma Congress, Brazil, 2022.

## Ethics Statement

This case report was prepared in accordance with the CARE Case Report Guidelines, and a completed CARE checklist is provided as Supporting Information (Available here).

## Consent

Written consent for publication of clinical details and images could not be obtained from the patient or his legal guardian because the patient was lost to follow‐up despite multiple attempts to contact them. All identifying information was removed, and only anonymized images are included in the manuscript. According to our institutional policy, formal approval from the Ethics Committee/Institutional Review Board was waived for this single anonymized case report.

## Conflicts of Interest

The authors declare no conflicts of interest.

## Supporting information


**Supporting Information** Additional supporting information can be found online in the Supporting Information section. CARE Checklist. Completed CARE checklist for this case report, indicating where each recommended reporting item is addressed in the manuscript.

## Data Availability

The data that support the findings of this study are available on request from the corresponding author. The data are not publicly available due to privacy or ethical restrictions.
