# Supplementary material for: Isolated Unilateral Posterior Sacroiliac Dislocation With an Intact Anterior Pelvic Ring: Case Report of an Unusual Injury Pattern
Source: Case Rep Orthop. 2026 Jun 22;2026:9605757. doi: 10.1155/cro/9605757 (PMC13285586; doi:10.1155/cro/9605757)
Supplement: Supplementary file 1 — Supporting Information Additional supporting information can be found online in the Supporting Information section. CARE Checklist. Completed CARE checklist for this case report, indicating where each recommended reporting item is addressed in the manuscript. [file CRO-2026-9605757-s001.pdf]

CARE case report guidelines

CARE Checklist of information to include when writing a case report

(cc) BY-NC-ND

Topic	Item	Checklist item description	Reported on Line
Title	1	The diagnosis or intervention of primary focus followed by the words "case report"	Lines 1-2
Key Words	2	2 to 5 key words that identify diagnoses or interventions in this case report, including "case report"	Line 25
Abstract (no references)	3a	Introduction: What is unique about this case and what does it add to the scientific literature?	Lines 30-37
	3b	Main symptoms and/or important clinical findings	Lines 34-37
	3c	The main diagnoses, therapeutic interventions, and outcomes	Lines 36-39
	3d	Conclusion—What is the main "take-away" lesson(s) from this case?	Lines 39-42
Introduction	4	One or two paragraphs summarizing why this case is unique (**may include references**)	Lines 44-61
Patient Information	5a	De-identified patient specific information.	Lines 64-66
	5b	Primary concerns and symptoms of the patient.	Lines 67-80
	5c	Medical, family, and psycho-social history including relevant genetic information	Lines 65-66
	5d	Relevant past interventions with outcomes	Line 66
Clinical Findings	6	Describe significant physical examination (PE) and important clinical findings.	Lines 77-85
Timeline	7	Historical and current information from this episode of care organized as a timeline	Lines 63-119
Diagnostic Assessment	8a	Diagnostic testing (such as PE, laboratory testing, imaging, surveys).	Lines 68-74; 89-99
	8b	Diagnostic challenges (such as access to testing, financial, or cultural)	Lines 75-77; 163-165; 194-201
	8c	Diagnosis (including other diagnoses considered)	Lines 71-73; 85-88
	8d	Prognosis (such as staging in oncology) where applicable	Not applicable
Therapeutic Intervention	9a	Types of therapeutic intervention (such as pharmacologic, surgical, preventive, self-care)	Lines 89-103; 112-115
	9b	Administration of therapeutic intervention (such as dosage, strength, duration)	Lines 100-115
	9c	Changes in therapeutic intervention (with rationale)	Not applicable
Follow-up and Outcomes	10a	Clinician and patient-assessed outcomes (if available)	Lines 109-117
	10b	Important follow-up diagnostic and other test results	Lines 104-108; 115-117
	10c	Intervention adherence and tolerability (How was this assessed?)	Lines 112-119
	10d	Adverse and unanticipated events	Lines 109-112; 115-117; 203-209
Discussion	11a	A scientific discussion of the strengths AND limitations associated with this case report	Lines 194-212
	11b	Discussion of the relevant medical literature **with references**.	Lines 121-158; 186-193
	11c	The scientific rationale for any conclusions (including assessment of possible causes)	Lines 159-193
	11d	The primary "take-away" lessons of this case report (without references) in a one paragraph conclusion	Lines 213-218
Patient Perspective	12	The patient should share their perspective in one to two paragraphs on the treatment(s) they received	Not available - lost to follow-up
Informed Consent	13	Did the patient give informed consent? Please provide if requested	**Yes** ☐ **No** ☒
